# HOSPITAL PHYSICAL THERAPY MANAGEMENT IN PEDIATRIC PATIENTS WITH COVID-19: CASE REPORTS

**DOI:** 10.1590/1984-0462/2021/39/2020238

**Published:** 2020-11-16

**Authors:** Camila Wohlgemuth Schaan, Vanessa de Souza Vieira, Cristina Miller, Ana Paula Dattein Peiter, Taciana Piccoli, Gabriela Cavion, Janice Luisa Lukrafka, Renata Salatti Ferrari

**Affiliations:** a Hospital de Clínicas de Porto Alegre, Porto Alegre, RS, Brazil.; b Universidade Federal de Ciências da Saúde de Porto Alegre, Porto Alegre, RS, Brazil.

**Keywords:** Coronavirus infections, Hospitalization, Pediatrics, Physical therapy, Infecções por coronavírus, Hospitalização, Pediatria, Fisioterapia

## Abstract

**Objective:**

To report the physiotherapeutic management of two pediatric cases with COVID-19 admitted in a reference state hospital to treat the disease in Porto Alegre, Southern Brazil.

**Cases description:**

*Case 1*, female, 10-month-old child, pre-existing chronic disease, hospitalized since birth, mechanical ventilation dependency via tracheotomy, progressed with hypoxemia, requiring oxygen therapy, and increased ventilator parameters, and a diagnosis of COVID-19 was confirmed. Airway clearance and pulmonary expansion maintenance therapies were performed. During hospitalization, the child acquired cephalic control, sitting without support, rolling, holding, and reaching objects. Recommendations were provided to a family member to maintain motor development milestones. *Case 2*, male, nine years old, previous psychiatric disease and obesity, showed worsening of the sensory state, requiring intensive care and invasive mechanical ventilation, with the diagnosis of SARS-Cov-2 infection. The physical therapy was performed to maintain airway clearance, pulmonary expansion, and early mobilization, showing ventilatory improvement during the intensive care hospitalization and successfully extubated after 17 days. The physical therapy evolved from passive to resistive exercises during the hospitalization, and the patient was able to walk without assistance at discharge, with the same previous functional status.

**Comments:**

The COVID-19 showed different manifestations in both cases. Physical therapy treatment was essential to maintain and to recover the functional status of the patients. Future studies are needed to improve the understanding of disease course and its functional consequences to offer an efficient treatment to pediatric patients with COVID-19.

## RESUMO

**Objetivo:**

Relatar as condutas fisioterapêuticas dos dois casos de pacientes pediátricos com COVID-19 internados em hospital de referência estadual em Porto Alegre para tratamento da doença.

**Descrição dos casos:**

Caso 1, sexo feminino, 10 meses de idade com doença crônica preexistente, internada desde o nascimento, utilizava ventilação mecânica via traqueostomia, evoluiu com hipoxemia, necessidade de oxigenoterapia e aumento dos parâmetros ventilatórios, sendo confirmada COVID-19. Foram realizadas técnicas de desobstrução brônquica e manutenção da expansão pulmonar. Além disso, a criança durante a internação adquiriu controle cefálico, sedestação sem apoio, rolar e alcance de objetos e durante infecção por coronavírus foram passadas orientações ao familiar para manutenção dos marcos motores adquiridos. Caso 2, sexo masculino, 9 anos, com doença psiquiátrica prévia e obesidade, evoluiu com quadro de rebaixamento do sensório e necessidade de tratamento intensivo, sendo o paciente colocado em ventilação mecânica invasiva na chegada à unidade e confirmada a infecção por SARS-CoV-2. Realizou fisioterapia para desobstrução brônquica, reexpansão pulmonar e mobilização precoce, apresentando melhora ventilatória ao longo da internação, e após 17 dias foi extubado com sucesso. Evoluiu de cinesioterapia passiva para assistida e resistida na internação pediátrica, conseguindo deambular sem auxílio, e teve alta hospitalar com condição funcional prévia à internação hospitalar.

**Comentários:**

A COVID-19 apresentou-se de forma distinta nos casos, todavia a fisioterapia foi essencial para a manutenção e recuperação do quadro funcional dos pacientes. Estudos futuros são necessários para melhor compreensão do curso da doença e suas repercussões funcionais, a fim de traçar um tratamento eficiente para os pacientes pediátricos acometidos pela COVID-19.

## INTRODUCTION

In December 2019, patients with pneumonia of unknown etiology were linked to a seafood and live animal market in the city of Wuhan, China.^1^ The new coronavirus is the seventh member of the family of coronaviruses that infect human beings ^1^ and whose disease was called COVID-19.^2^ With rapid spread, it was declared by the World Health Organization (WHO) as a pandemic,^2^ with its first case confirmed in Brazil on February 26, 2020^3^ and in Rio Grande do Sul on March 10, 2020.^4^

Reports indicate an aggressive pattern of COVID-19, especially in the older population and with comorbidities when compared to younger people.^5^ However, in an Italian study, 65.1% of the pediatric cases analyzed required hospitalization in Italy^6^ ^,^ and 5% required intensive care in Madrid, Spain.^7^ Clinical manifestations include fever, dry cough and fatigue,^8^ and more than 90% do not progress to the severe form of the disease.^9^ Nevertheless, factors such as younger age, underlying lung disease, and immunosuppression are not ruled out as predisposing to more severe cases of COVID-19 in children.^8^

Physical therapy is among the resources used in moderate to severe cases.^10^ To date, we do not know of any studies reporting physical therapy management in pediatric patients with COVID-19 or the functional repercussions after hospitalization. Therefore, aiming at contributing with scientific evidence and an understanding on the progression and therapeutic process, the objective of the study was to report the two cases of pediatric patients with COVID-19 treated at Hospital de Clínicas de Porto Alegre (HCPA), state reference center in fighting the disease.

## CASE DESCRIPTIONS

### Case 1

A 10-month-old female patient admitted to HCPA for nine months, remained in the Neonatal Intensive Care Unit (NICU) for four months for genetic investigation, requiring invasive mechanical ventilation (IMV) to stabilize the respiratory condition. During hospitalization, she needed tracheostomy (TQT) and IMV for home use. For this purpose, at the NICU, the patient was also adapted to the Trilogy 100 mechanical ventilator (Philips Respironics, United States), pressure-controlled ventilation mode, inspiratory pressure (IPAP) of 25cmH_2_O, expiratory pressure (EPAP) of 6 cmH_2_O, respiratory rate (RR) of 22 ripm, auto-track sensitivity, and inspiratory time of 0.8 seconds.

After her adaptation, she was discharged to the pediatric inpatient unit, where she was clinically stable, not requiring oxygen therapy, awaiting approval by the legal authorities for *home care*. However, in the ninth month of hospitalization, she started with fever and hypoxemia [partial oxygen saturation (SpO_2_): 90%], requiring the placement of oxygen (2L/min), and the transfer to an isolation bed, in which she underwent polymerase chain reaction (PCR) test for COVID-19, whose result was positive.

The unit’s physical therapy team, to prevent aerosol dispersion, installed a closed suction circuit, although little effective, since it is an active child who frequently disconnected the IMV circuit. Also, a high-efficiency particulate air (HEPA) filter was inserted at the vent outlet, followed by a heat and moisture exchanger (HME) close to the TQT. The heat and moisture exchanger filter (HMEF) was used in the manual resuscitator to perform airway clearance techniques and a HEPA filter at the outlet of the compressed air, aiming at reducing aerosol dispersion during airway aspiration.

**Figure 1:**
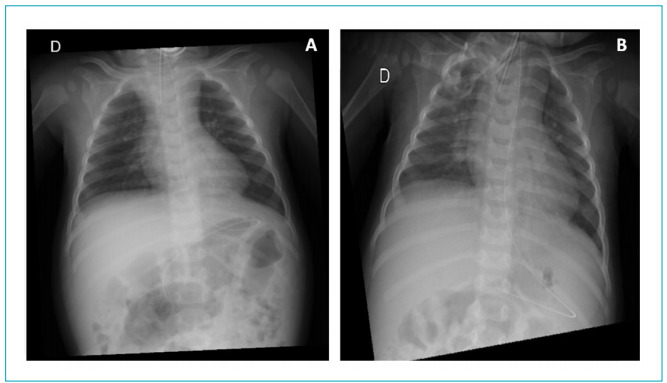
Chest radiography: (A) without changes; (B) perihilar infiltrate and apparent middle lobe consolidation.

On the initial chest X-ray, there were no changes (Figure 1A1). Venous blood gas analysis indicated the presence of respiratory acidosis (pH=7.27; pCO_2_=69.9; HCO_3_=31.7), requiring adjustments in ventilatory parameters (IPAP=28 cmH_2_O; EPAP=5 cmH_2_O; RR=25 ripm), and use of oxygen therapy (1 L/min). Seven days after the onset of symptoms, a new chest X-ray was performed (Figure 1B), with the presence of perihilar infiltrate and apparent middle lobe consolidation.

During physical therapy sessions, manual techniques were performed, aiming at maintaining pulmonary expandability and promoting bronchial hygiene. The most used techniques were: compression/decompression, prolonged slow expiration (PSE), manual hyperinflation (MH) with self-inflating bag and bag squeezing. To keep the patient active and prevent the regression of the motor condition, daily motor stimulation was maintained by guiding the relative.

Nineteen days after the onset of COVID-19 symptoms, the patient was discharged from the hospital, clinically stable, with negative control for SARS-CoV-2, blood gases without changes, and return to the ventilatory parameters used before the infection. A few days before discharge, a genetic investigation was completed, confirming Ondine’s syndrome. Despite being a prolonged hospitalization (300 days), the patient had cephalic control, sat without support, rolled, and performed holding and reaching objects without difficulty. In addition, functional assessment using the Functional Status Scale (FSS-Brazil) indicated a moderate degree of dysfunction (12 points), and the attendance of motor and respiratory physical therapy was continued at home.

### Case 2

**Figure 2:**
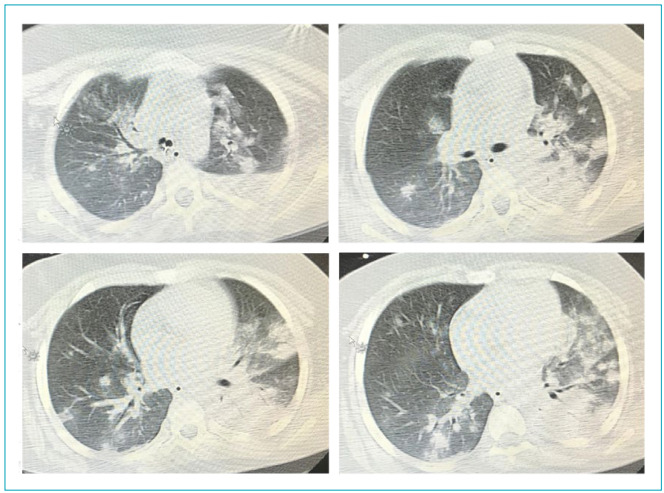
Chest tomography: associated with suspected changes for viral pneumonia, there are extensive consolidations in the left inner lobe and a small pleural effusion (bacterial infection associated with COVID-19 pneumonia?).

Male patient, nine years and 11 months old, previous diagnosis of autism spectrum disorder (ASD), oppositional behavior, bipolar affective disorder, cognitive deficit, difficult to control epilepsy, and obesity, hospitalized via emergency and transferred from the hospital in his city by suspected COVID-19. History of fever up to 39.5°C for four days associated with sporadic cough, hyaline secretion, convulsive escape, and reduced appetite and urinary volume. Throughout the day, on April 1, he presented worsening of the sensory part, being referred to the Pediatric Intensive Care Unit (PICU), with a Glasgow coma scale 3, miotic pupils, SpO_2_=80%, and immediate tracheal intubation was performed. Chest computed tomography compatible with the ground-glass standard was performed (Figure 2). SARS-CoV-2 testing was positive.

After four days, he started physical therapy three times a day, aiming at airway clearance, improvement of gas exchange and prevention of deleterious effects related to bed restriction, such as decreased range of motion and loss of muscle mass, with the following approaches: compression/decompression, aspiration of endotracheal tube and upper airways, passive exercise, limb stretching, and functional positioning in bed. On that same day, adjustments were made to the IMV to ensure a tidal air volume of 7.5 mL/kg, and the oxygen saturation index (OSI) was evaluated, resulting in 6.5, which characterize mild acute respiratory distress syndrome (ARDS).

**Figure 3:**
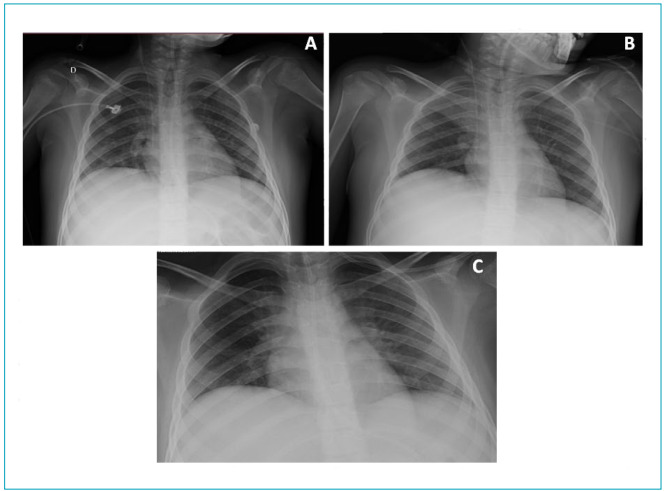
Chest radiography: (A) hypoexpanded lungs; (B) atelectasis of the right upper lobe and bilateral hypoexpansion; (C) hypoexpanded lungs.

After 14 days from the start of hospitalization, the patient showed a clinical and hemodynamic improvement, good response to diuretic therapy and prone position (performed four times during hospitalization), progressive ventilatory improvement, tolerating weaning from ventilation. He was extubated after 17 days of IMV. He continued with oxygen therapy via a non-rebreather mask (7 L/min), maintaining SpO_2_=96%, requiring frequent airway aspiration due to the accumulation of secretion and ineffective cough. In physical therapy, the maneuvers mentioned above were performed, as well as periods of non-invasive mechanical ventilation (NIMV), bilevel mode, IPAP = 14 cmH_2_O, EPAP=8 cmH_2_O, FiO_2_=40%, due to pulmonary hypoexpansion (Figure 3A), aspiration of airways, passive exercise evolving to assisted, stretching, and transfer training.

To prevent aerosol dispersion, aspiration during IMV occurred via a closed system and NIMV through a mechanical ventilator with double branch, total face interface (not ventilated), HEPA filter on the expiratory branch, and HME filter proximal to the patient. Over the days, the child tolerated oxygen reduction and transition to a nasal catheter at 3 L/min. He maintained a stable condition and progressive improvement, enabling discharge from the PICU after 22 days. At that time, he scored 17 on the FSS-Brazil, a score characterized as severe dysfunction.

The day after discharge from the ICU, in the pediatric inpatient unit, the patient developed drowsiness, hyporesponsiveness, and difficulty in managing airway secretion. A chest X-ray was performed (Figure 3B), showing atelectasis, and, besides the procedures described above, the MH technique with a self-inflating bag and HEPA filter (twice a day, three cycles of 10 repetitions each) was used to avoid aerosol dispersion. After three days, a chest X-ray was repeated with total resorption of atelectasis (Figure 3C), supplemental oxygen was removed, and the MH technique was suspended, with no need for airway aspiration. Pulmonary auscultation had no adventitious sounds. Also, according to the patient’s tolerance and collaboration, assisted and active exercise and transfer training were performed (orthostasis and ambulation in the room with assistance). It was possible to progress to resisted exercise and independent ambulation. Physical therapy took place twice a day.

The patient was discharged after 41 days of hospitalization with a negative PCR for SARS-CoV-2, 8 points in the FSS-Brazil, a score characterized as mild dysfunction, and 60 points in muscle strength as assessed by the Medical Research Council (MRC).

In both cases, the institution’s precautionary measures to care for suspected and confirmed patients with COVID-19 were used by professionals involved in care during hospitalization.

## DISCUSSION

The role of the physical therapist inserted in the multiprofessional team aims at treating the functional changes caused by COVID-19, assisting in the management of ventilatory support and in airway clearance to improve gas exchange and facilitate weaning from mechanical ventilation.^11^ Furthermore, due to the prolonged hospital stay, in critically ill patients with a long period on IMV, muscle weakness becomes a problem,^12^ and may manifest itself in young children with loss of motor milestones and delay in motor development.^13^

The assessment of global functionality was performed using the FSS-Brazil scale,^14^ ^,^ ^15^ based on which the patient in case 1 was discharged with moderate dysfunction, with the domains most affected being food and breathing, due to the use of nasoenteral tube and IMV. In case 2, the degree of dysfunction at discharge was mild due to the higher score in the food domain, and muscle strength was preserved. In this case, early mobilization was carried out since cardiorespiratory stabilization, aiming at preventing and/or minimizing loss of range of motion, muscle strength, and cardiorespiratory conditioning.^16^ Besides that, the exercises considered the patient’s previous functional condition, with progressively increasing intensity and frequency, according to the patient’s clinical condition and tolerance.^17^

The breathing techniques used followed the guidelines,^18^ age, and patient collaboration. Although most cases of COVID-19 evolve with a dry cough,^19^ in both cases described there was impairment of bronchial hygiene, and techniques were applied for this purpose. In case 1, aspiration was performed through an open system, due to the non-adaptation of the tracheostomy to the closed system. All the institution’s precautionary measures for procedures that generate aerosol in suspected or confirmed cases of COVID-19 were taken by the professionals involved, with no contamination by the team. In case 2, the manual hyperinflation technique with self-inflating bag and HEPA filter, followed by chest compression maneuvers, was used in the pediatric inpatient unit, as it is an airway clearance technique and pulmonary reexpansion alternative to NIMV, since the devices available in wards have single-branches, with a valve interface and consequent aerosol elimination, which should be avoided.^20^

Oxygen therapy has been indicated in cases of hypoxemia (SpO_2_<94%).^10^ ^,^ ^21^ In case 1, supplementation was performed using a T-ayre piece via TQT to maintain SpO_2_ levels > 90%, as this is a patient with previous chronic disease. In case 2, a non-rebreather mask was used for oxygen flows above 7 L/min and a nasal catheter for smaller flows.^10^ ^,^ ^21^ Concerning the IMV circuit, in case 1, to reduce aerosol dispersion, the patient used a single circuit. Therefore, the indication is for an HMEF filter proximal to TQT (before the exhalation valve).^22^ However, the patient presented a decrease in the volume of tidal air and a consequent decrease in saturation (SpO_2_~82%), and it was necessary to use only a proximal HME filter and HEPA filter at the ventilator outlet, avoiding contamination. In case 2, the NIMV circuit followed the guidelines^20^ ^,^ ^23^ and, despite the controversies about its use in patients with COVID-19, as the objective was therapeutic (pulmonary reexpansion), it was well tolerated and used successfully.

As a conclusion, pediatric patients affected by COVID-19 presented different forms of disease manifestation, and in both cases, physical therapy was essential for maintaining and improving the functional status. The use of non-invasive ventilation, although still controversial, was essential for maintaining and improving the respiratory condition. Also, precautions for confirmed and suspected cases of the disease were taken by all professionals. Future studies are needed to support physiotherapeutic approaches, as COVID-19 is a new disease, not very prevalent among children, making its understanding and its outcomes in this population a challenge.
